# Drivers of Active Amphetamine-Type Stimulant Use among Transgender Women in Malaysia: A Cross-Sectional Study

**DOI:** 10.21203/rs.3.rs-3607148/v1

**Published:** 2023-11-16

**Authors:** Luzan JadKarim, Jonathan Galka, Roman Shrestha, Rosanna Mazzeo, Arjee Restar, Kamal Gautam, Iskandar Azwa, Jeffrey A. Wickersham

**Affiliations:** Yale University; Harvard University; University of Connecticut; Yale University; University of Washington; University of Connecticut; University of Malaya; Yale University

**Keywords:** ATS, transgender women, sex work

## Abstract

While ample evidence exists regarding the use of active amphetamine-type stimulants (ATS) among sex workers, the impact of ATS use has yet to be characterized among the transgender population in Malaysia. Our aim is to highlight and assess health-related factors associated with ATS use among transgender women in Malaysia. A total of 361 transgender women completed a cross-sectional survey regarding their attitude towards PrEP knowledge and use for HIV prevention. The original study explored a myriad of health-related topics including active ATS use. Data was analyzed using logistic regression analyses to determine factors associated with active ATS use. Most of the participants were between 25–40 years old (57.3%), ethnically identified as Malay (75%), and single (67.6%). We found that 10.2% of the participants were actively using ATS. On a multivariate level, hormone therapy use was associated with reduced odds of active ATS use (aOR = 0.364; 95% CI = 0.169, 0.784) and was positively associated with a history of drug related arrest (aOR = 4.604; 95%CI = 1.813, 11.691). Our findings show a high prevalence of active ATS use among transgender women in Malaysia, in addition to its correlation to other health- related factors. Interestingly, we found that trans women who were actively using hormone therapy, were less likely to engage in active ATS use. This relationship should be explored further along with the relationship between incarceration history. In addition, further prevention strategies and efforts are needed to decrease ATS use among transgender women in Malaysia.

## Introduction

Amphetamine-type stimulants (ATS) are synthetic psychostimulants that upon intake can stimulate the central nervous system, allowing users to experience excitation, arousal, euphoria, and increased alertness through the release dopamine, adrenaline, noradrenalin, and serotonin (1, 2). Some examples of ATS include methamphetamine, amphetamine and ecstasy; they can be injected, smoked, or taken orally (3). Both transgender and cis-gender women, involved in sex work will make use of ATS with clients to improve sexual desire and behavior in addition to increasing functionality and stamina when working long hours (3). However, the repeated use of the stimulants leads to addiction.

Transgender women (TW) are defined as individuals who were assigned to the male gender at birth, but identify as female (4). Globally, TW face one of the highest HIV burdens, thus experiencing an elevated risk of contracting HIV (5). Previous findings show an increased risk among TW in the Asia-Pacific regions, especially in Malaysia (6). Furthermore, the prevalence of HIV is four times higher among TW who engage in sex work when compared to female sex workers due to anal intercourse increasing the probability of HIV contraction (5). ATS use is common among female sex workers and has been associated with higher risk of HIV infection (3).

The use of ATS can be linked to increased risk of HIV, especially when injection is involved. ATS can cause impaired judgment resulting in unsafe sexual practices, another risk factor (7, 8). Women involved in sex work begin using ATS to please their clients, however, it often turns into a habit following its initial use. The combination of sexual health risk behaviors including active ATS use amplifies the odds of contracting HIV among other sexually transmitted illnesses (STI). ATS use has been a growing issue in Malaysia, putting individuals at a higher risk for contracting HIV, especially among marginalized populations like female sex workers and TW (8).

The correlation between ATS use and increased risk of HIV has been explored wherein ATS use increased the likelihood of risky sexual practices and the number of sex partners, thus increasing the risk of HIV for individuals who actively use it (1). Intoxicated clients of sex workers often request that condoms not be utilized; cis and trans-gender sex workers comply with these requests in order to please their clients, which can lead to a higher probability of contracting HIV (1). Clients who are looking to engage in unprotected sex will seek out sex workers who utilize drugs with the knowledge that they will most likely comply with their requests, as their decision-making skills may be impaired when under the influence. Clients will also frequently offer sex workers ATS to ensure they receive the services they desire (1). Similarly, research has shown that clients who want to participate in unprotected sex are generally intoxicated from ATS (1).

Using data from a cross-sectional survey conducted in Malaysia, we sought to gain a better understanding on active ATS use among TW. Correlates of ATS use within the past 30 days were examined to further study their relationship with different risk behaviors. We aimed to highlight and assess health-related factors associated with ATS use among TW in Malaysia to better understand challenges related to substance use and HIV prevention efforts and strategies.

## Methods

### Study Design and Sample.

Using a cross-sectional study design, we asked TW in Malaysia to answer a survey that evaluated their attitudes towards PrEP and other health-related issues including their ATS use. The eligibility criteria included: 1) 18 years of age or older, 2) assigned male sex at birth and self-identify as female or TW, 3) living in Malaysia, and 4) able to speak or read Bahasa Malaysia, Tamil, or English.

### Recruitment.

Initially 381 participants were screened for the study, with 7 being ineligible and 13 refusing to participate. As a result, a total of 361 TW completed the study. Participants were recruited via convenience sampling wherein flyers were posted in areas frequented by TW, as well as social media, and referrals from staff at community-based organizations serving TW. Participants were recruited from three states in West Malaysia including Selangor, Penang, and Negeri Sembilan, as well as Kuantan and Pahang in East Malaysia. After providing consent, participants partook in self-administered survey on the Qualtrics software. The survey took approximately 20 minutes to complete. Once completed, the participants were allowed to ask any additional questions regarding the research study and were compensated with 20 Malaysian Ringgit (~ 4 USD) for their time. Full methods are provided in (9).

### Study Ethics.

This study was approved by the Institutional Review Board of Yale University and the University of Malaysia.

### Consent.

Participants provided informed consent prior to enrolling in the study.

#### Measures.

##### Dependent Variable.

The variable of interest, active ATS use, was measured by a combination of two single-item questions, “In the past 30 days, have you used crystal methamphetamine (ice, syabu)?” and “In the past 30 days, have you used ecstasy (E, Pil kuda, MDMA)?”, with a dichotomous response of “yes” or “no” for each.

##### Participant Characteristics.

Variables that described the sociodemographic characteristics of the participants were collected, including age, ethnicity, monthly income, education status (defined as obtained a high school diploma), living in stable housing (defined as residing in a house or apartment alone or with others), religion, relationship status, and mental health status (moderate to severe depressive symptoms). Participant ethnicity was coded per the largest ethnic backgrounds in Malaysia, hence the four categories of Malay, Chinese, Indian, or other. Monthly income was collected as continuous and was coded into three categories to represent the at or below poverty level (≤1169 RM), low income (1170–2208 RM), and middle-upper income (≥2209 RM) ranges per recent income statistics in Malaysia (10). Since Islam is the primary religion in Malaysia, we coded the religion variable to reflect Muslims and those who were not Muslims. The remaining sociodemographic variables mentioned were also dichotomous.

##### Sexual Health History.

The sexual health history variables included recent doctor visit, lifetime STI diagnosis, active hormone therapy use, sexual assault history, physical violence history, and active use of cuci darah. Cuci darah, which when translated from Bahasa Malaysia to English means “blood washing,” and refers to the self-use of unregulated oral, gel capsules that are perceived to contain antibiotics often obtained in the community from peers. All sexual health history questions were measured dichotomously.

##### Sexual Risk Behavior.

Participants were asked questions regarding their sexual risk behavior including: condomless sex with casual partners (defined as a person who is not the primary partner and who does not pay for sex), condomless sex with primary partner (defined as the who does not pay for sex), their sex work status (defined as the frequency of engagement in transactional sex), their involvement in sex work pre-18 years, and their primary method of client solicitation (online vs street-based). All variables were dichotomous except for sex work status which included three categories no sex work, part-time, and full-time statuses.

##### Criminal Justice Involvement.

Variables regarding participants’ criminal history included previous incarcerations, history of drug related arrests, and history of sex work related arrests (defined as having ever been arrested for sex work). All variables were kept as dichotomous.

##### Statistical Analysis.

Of the 381 survey responses, 361 were determined as eligible and therefore were analyzed. A bivariate logistic analysis was conducted on various measures including participant characteristics, sexual health history, and sexual risk behavior variables with the results listed in table 1. Those that were found to be significantly (p<0.1) associated with active ATS use from table 1 were included in the multivariate analysis presented in table 2. Multicollinearity between independent variables was tested using the variance inflation factor (VIF) with the cutoff being at 2.4. Significantly associated (p<0.05) variables from table 2 were then reported and discussed further. The software used for data analyses was IBM SPSS Statistics for Windows version 28.00.

## Results

A bivariate analysis was initially conducted on study measures to determine which variables were associated with active ATS use among transgender Malaysian women, all reported in Table 1. Approximately 10% of the participant in this study have reported using ATS in the past 30 days. The mean age for TW was 35 years (SD = 9.81), they were ethnically Malay (75%), and identified as Muslim (79.8%). The majority of the participants did have the equivalent of a high school diploma (69.3%) and were living in stable housing (95%).

In the bivariate logistic regression active ATS use was associated with being Malay (OR = 2.276, 95% CI = 0.859, 6.031), being single (OR = 2.203, 95% CI = 0.938, 5.176), actively using cuci darah (OR = 2.374, 95% CI = 0.958, 5.878), having full-time sex work status (OR = 2.978, 95% CI = 1.069, 8.295), having a history of drug related arrest (OR = 5.965, 95% CI = 2.781, 12.797), and having a history of sex work related arrest (OR = 1.793, 95% CI = 0.905, 3.554). Active ATS was also associated with active HT use (OR = 0.347, 95% CI = 0.169, 0.715) but inversely.

Results of the multivariate variable analysis are reported in Table 2. History of drug related arrest was significantly associated with more than four-fold increase of active ATS use (aOR = 4.604, 95% CI = 1.813, 11.691). On the other hand, participants who were actively using HT had a significantly reduced odds of active ATS use by (aOR = 0.364, 95%CI = 0.169, 0.784).

## Discussion

The literature surrounding active ATS use among Malaysian TW is very limited. Our study is one of the few that reports on the correlates of active ATS use among TW and adds to the scant evidence surrounding this key population. Our findings indicate that 1 in 10 TW in Malaysia actively use ATS, which is consistent with studies of TW in other countries of Southeast Asia (7).

Malaysia has become an increasingly significant gateway for global drug trafficking, and recent reports indicate an increase in ATS-related arrests as Malaysian authorities increase enforcement (11). Therefore, it was no surprise when we found that participants who were arrested for drug charges had over four times the odds of also actively using ATS. Recent findings also show that individuals who were previously incarcerated in Malaysia for drug charges had higher likelihood of testing positive for drug use after serving their time (12). Our results provide further evidence to the association between active ATS use and the criminal justice involvement, suggesting that drug rehabilitation among prisoners may aid in decreasing the likelihood of drug relapse post release.

Other literature postulates that sex workers, including TW, who use ATS also reported recent involvement in sex work, which was reflected in our results as well as others (13). Prior research posits that ATS use contributes to impaired decision-making around sexual health such as condom use, PrEP adherence, increased sex partners, and elevated exposure to sexually transmitted illnesses (STI) among marginalized populations including TW (1, 13–16). As such, we found that participants who were involved in sex work fulltime and had previous arrests relating to sex work experienced elevated odds actively using ATS on a bivariate level. We urge for further research on the relationship between sex work and active drug use, including ATS, among Malaysian TW is necessary to helping the population decrease ATS uptake.

The role of HT and drug use among TW has not been studied extensively. Previous findings posit that substance use, including ATS use, increases when TW experience stress, anxiety, depression, and overall negative emotions due to unmet gender affirming care (GAC) needs (17–19). We found that TW who were receiving HT had a reduced odds of active ATS use, making HT a preventative factor. This finding is supported by a secondary analysis that found TW in San Francisco who utilized GAC had significantly lower odds of using noninjectable drugs including ATS (20). Since non-prescribed drug use and abuse have been associated with poor mental health and low self-esteem among transgender adults, having access to and utilizing GAC, including HT, may alleviate these internal stressors thus reducing their vulnerability to substance abuse (17–20). In our findings, TW who were receiving GAC, in the form of HT, have lower levels of unmet care needs and/or lower levels of stress, anxiety, and other negative emotions and therefore had lower odds of actively using ATS. Perceived discrimination, both physical and mental, has been linked to poor mental health and suicidal ideations among TW, having access to GAC may ameliorate these negative emotions and possible cognitive dissonance among TW (20–22). In addition, TW who utilize GAC, including HT, have reported lower odds of having suicidal ideations and higher odds of STI testing showing better care for their sexual health (20, 23). Therefore, efforts to improve access to HT and other forms of GAC to the transgender community in Malaysia are crucial to preventing further substance use and abuse in addition to improving the population’s general health outcomes.

One notable finding that, to the best of our knowledge, has not been examined is the association of cuci darah and active ATS use. On a bivariate level, we found that Malaysian TW had higher odds of actively using ATS when they were also actively using cuci darah. However, the multivariate analysis showed no significant results. Translating to blood wash, cuci darah is a term coined among the TW Malaysian population to describe the prophylactic use of oral medications, most likely antibiotics, to prevent STIs. Given their prominent use among the transgender community in Malaysia, we urge further research into their use and impact on healthcare outcomes of transgender adults.

## Strengths and Limitations

TW are a marginalized subpopulation in Southeast Asia, especially in largely religious countries like Malaysia. Our study presents various notable strengths including being one of the only studies to address the correlates of active ATS use among Malaysian TW. In addition, our findings highlight the need to investigate and understand the use of HT among TW in a culture that largely does not acknowledge their issues. Furthermore, the use of cuci darah among Malaysian TW has not been documented or studied and our study is the first to report its use in relation to ATS use.

This study is not without limitations. Firstly, the cross-sectional nature of the study does not allow for temporal or causal relationships to be established between active ATS and the related covariates. Secondly, the generalizability of our findings only applies to TW in Malaysia and may not extent to other TW in the region. Moreover, the study did utilize venue-based and snowball sampling, which is another limitation towards generalizability of the findings. Finally, the study when conducted was exploratory in nature and was not specified for ATS users. We recommend that future research be conducted using random sampling methods such that the participant pool is more diverse and a more specific approach towards ATS users.

Despite the listed limitations, efforts to acknowledge and understand the role of ATS among marginalized populations in Malaysia are critical. Historically, harm reduction efforts have largely ignored transgender people who use drugs. The current dominant effort to reduce ATS use in Southeast Asia is incarceration and heavy policing, and both have been shown to be harmful and ineffective (12, 24). Prevention efforts to decrease ATS, such as HT use, among TW need expansion; efforts ranging from educational programs that raise the awareness of ATS use and risk, substitution treatment for amphetamine dependence using sustained-release dexamphetamine, and distribution of safe smoke kits and safe sex products should be implemented throughout safe-spaces and clinics for TW in Malaysia (24, 25). Additionally, more social support should be made available to TW within the healthcare field to allow them access to much needed treatment and therapies.

### Conclusion.

We found that active ATS use among TW in Malaysia to be associated with their criminal justice involvement. In addition, we found that GAC efforts, such as HT use, are also associated with active ATS use and in fact a preventative factor among this under studied population. For future directions we suggest that efforts to target and understand the transgender community and their hurdles to healthcare in Malaysia need to increase. Moreover, the ATS crisis and drug related arrests in Malaysia should further be studied considering that the country is a transit in drug trafficking. Finally, we implore healthcare providers to implement prevention strategies that decrease active ATS use, HIV infections, and substance use among TW in Malaysia.

## Figures and Tables

**Table 1.** Bivariate Factors Associated with Active Amphetamine-Type Stimulant Use Among Transgender Women (n=361).

Variable	N = 361	Active ATS Use n (%)		OR (95% CI)	p-value
		Yes 37 (10.2)	No 324 (89.8)		
**Participant Characteristics**					
Age					
18 – 25 years	63 (17.5)	5 (13.5)	58 (17.9)	1.395 (0.521, 3.735)	0.507
25 – 40 years	207 (57.3)	22 (59.5)	185 (57.1)	0.907 (0.454, 1.813)	0.783
41+ years	91 (25.2)	10 (27)	81 (25)	0.900 (0.418, 1.940)	0.788
Ethnicity					
Malay	271(75)	32 (86.5)	239 (73.7)	**2.276 (0.859, 6.031)**	**0.098**
Chinese	9 (2.5)	0 (0)	9 (2.8)	0.000 (0.000)	0.999
Indian	48 (13.3)	4 (10.8)	44 (13.6)	0.771 (0.261, 2.283)	0.639
Other	33 (9.2)	1 (2.7)	32 (9.9)	0.253 (0.034, 1.911)	0.183
Monthly income					
<1169 RM	93 (25.8)	8 (21.6)	85 (26.2)	0.776 (0.341, 1.763)	0.544
1170 – 2208 RM	111 (30.7)	12 (32.4)	99 (30.6)	1.091 (0.527, 2.259)	0.815
>2209 RM	157 (43.5)	17 (46)	140 (43.2)	1.117 (0.564, 2.212)	0.751
High school diploma					
No	111 (30.7)	13 (35.1)	98 (30.2)	REF	REF
Yes	250 (69.3)	24 (64.9)	226 (69.8)	0.801 (0.391, 1.637)	0.542
Stable housing					
Stable	343 (95)	35 (94.6)	308 (95)	REF	REF
Unstable	18 (5)	2 (5.4)	16 (5)	1.100 (0.243, 4.984)	0.902
Religion					
Other	73 (20.2)	6 (16.3)	67 (20.6)	REF	REF
Muslim	288 (79.8)	31 (83.7)	257 (79.4)	1.347 (0.540, 3.362)	0.523
Relationship status					
Partner	117 (32.4)	7 (19)	110 (34)	REF	
Single	244 (67.6)	30 (81)	214 (66)	**2.203 (0.938, 5.176)**	**0.070**
Moderate to severe depressive symptoms					
No	159 (44)	13 (35.1)	146 (45)	REF	REF
Yes	202 (56)	24 (64.9)	178 (55)	1.514 (0.745, 3.079)	0.252
**Sexual Health History**					
Active cuci darah use (30 days)					
No	325 (90)	30 (81)	295 (91)	REF	REF
Yes	36 (10)	7 (19)	29 (9)	**2.374 (0.958, 5.878)**	**0.062**
Seen a doctor in past 12 months					
No	54 (15)	7 (19)	47 (14.5)	REF	REF
Yes	307 (85)	30 (81)	277 (85.5)	0.727 (0.302, 1.751)	0.477
Ever diagnosed with an STI					
No	326 (90.3)	31 (83.7)	295 (91)	REF	REF
Yes	35 (9.7)	6 (16.3)	29 (9)	1.969 (0.759, 5.110)	0.164
Active hormone therapy use (90 days)					
No	161 (44.6)	25 (67.6)	136 (42)	REF	REF
Yes	200 (55.4)	12 (32.4)	188 (58)	**0.347 (0.169, 0.715)**	**0.004**
Childhood sexual assault					
No	212 (58.7)	21 (56.8)	191 (59)	REF	REF
Yes	149 (41.3)	16 (43.2)	133 (41)	1.094 (0.550, 2.175)	0.797
Adulthood sexual assault					
No	283 (78.4)	26 (70.2)	257 (79.3)	REF	REF
Yes	78 (21.6)	11 (29.8)	67 (20.7)	1.623 (0.763, 3.451)	0.208
Childhood physical violence					
No	226 (62.6)	23 (62.2)	203 (62.7)	REF	REF
Yes	135 (37.4)	14 (37.8)	121 (37.3)	1.021 (0.556, 2.060)	0.953
Adulthood physical violence					
No	239 (66.2)	27 (73)	212 (65.4)	REF	REF
Yes	122 (33.8)	10 (27)	112 (34.6)	0.701 (0.328, 1.500)	0.360
**Sexual Risk Behavior**					
Condomless sex with casual partner (30 days)					
No	278 (77)	27 (73)	251 (77.5)	REF	REF
Yes	83 (23)	10 (27)	73 (22.5)	1.273 (0.589, 2.753)	0.539
Condomless sex with partner (30 days)					
No	270 (74.8)	26 (70.2)	244 (75.3)	REF	REF
Yes	91 (25.2)	11 (29.8)	80 (24.7)	1.290 (0.610, 2.729)	0.505
Sex Work Status					
None	92 (25.4)	5 (13.5)	87 (26.8)	REF	REF
Part-time	139 (38.5)	13 (35.2)	126 (38.9)	1.795(0.618, 5.218)	0.282
Full-time	130 (36)	19 (51.3)	111 (34.3)	**2.978 (1.069, 8.295)**	**0.037**
Online sex work solicitation					
No	208 (57.6)	20 (54)	188 (58)	REF	REF
Yes	153 (42.4)	17 (46)	136 (42)	1.175 (0.593, 2.327)	0.644
Street-based sex work solicitation					
No	186 (51.5)	17 (46)	169 (52)	REF	REF
Yes	175 (48.5)	20 (54)	155(48)	1.283 (0.648, 2.538)	0.474
Sex work involvement pre-18					
No	252 (69.8)	24 (64.9)	228 (70.4)	REF	REF
Yes	109 (30.2)	13 (35.1)	96 (29.6)	1.286 (0.629, 2.632)	0.490
**Criminal Justice Involvement**					
Previously incarcerated					
No	284 (78.6)	23 (62.2)	261 (80.5)	REF	REF
Yes	77 (21.3)	14 (37.8)	63 (19.5)	**2.522 (1.229, 5.176)**	**0.012**
History of drug related arrest					
No	317 (87.8)	23 (62.2)	294 (90.7)	REF	REF
Yes	44 (12.2)	14 (37.8)	30 (9.3)	**5.965 (2.781, 12.797)**	**<0.001**
History of sex work related arrest					
No	231 (64)	19 (51.4)	212 (65.4)	REF	REF
Yes	130 (36)	18 (48.6)	112 (34.6)	**1.793 (0.905, 3.554)**	**0.094**

Numbers that are in boldface indicate significance of p<0.10.

REF denotes reference group.

**Table 2.** Factors Associated with Active Amphetamine-Type Stimulant Use Among Transgender Women in a Multivariate Logistic Model.

Variable	B	aOR (95% CI)	p-value
Ethnicity			
Malay	0.976	2.653 (0.923, 7.628)	0.070
Relationship Status			
Single	0.354	1.424 (0.574, 3.535)	0.446
Active cuci darah use (30 days)			
Yes	0.808	2.243 (0.788, 6.388)	0.130
Active hormone therapy use (90 days)			
Yes	−1.012	**0.364 (0.169, 0.784)**	**0.010**
Sex Work Status			
None	REF	REF	REF
Part-time	0.193	1.213 (0.372, 3.955)	0.749
Full-time	0.848	2.335 (0.743, 7.346)	0.147
Previously incarcerated			
Yes	0.256	1.292 (0.533, 3.133)	0.571
History of drug related arrest			
Yes	1.527	**4.604 (1.813, 11.691)**	**0.001**
History of sex work related arrest			
Yes	0.036	1.037 (0.470, 2.289)	0.929

Numbers that are in boldface indicate significance of p<0.05.

REF denotes reference group.
